# Separation of the Sound Power Spectrum of Multiple Sources by Three-Dimensional Sound Intensity Decomposition

**DOI:** 10.3390/s21010279

**Published:** 2021-01-04

**Authors:** Shiyi Chai, Xiaoqin Liu, Xing Wu, Yanjiao Xiong

**Affiliations:** 1 School of Key Laboratory of Vibration and Noise under Ministry of Education of Yunnan Province, Kunming University of Science and Technology, Kunming 650500, China; chaishiyi@stu.kust.edu.cn (S.C.); xwu@kust.edu.cn (X.W.); yan.jiao.ok@163.com (Y.X.); 2 Yunnan Vocational College of Mechanical and Electrical Technology, Kunming 650203, China

**Keywords:** three-dimensional sound intensity, spectrum separation, sound power, particle swarm optimization, condition monitoring

## Abstract

The identification and separation of sources are the prerequisite of industrial noise control. Industrial machinery usually contains multiple noise sources sharing same-frequency components. There are usually multiple noise sources in mechanical equipment, and there are few effective methods available to separate the spectrum intensity of each sound source. This study tries to solve the problem by the radiation relationship between three-dimensional sound intensity vectors and the power of the sources. When the positions of the probe and the sound source are determined, the sound power of the sound source at each frequency can be solved by the particle swarm optimization algorithm. The solution results at each frequency are combined to obtain the sound power spectrum of each sound source. The proposed method is first verified by a simulation on two point sources. The experiment is carried out on a fault simulation test bed in an ordinary laboratory; we used three three-dimensional sound intensity probes to form a line array and conducted spectrum separation of the nine main noise sources. The sound intensity on the main frequency band of each sound source was close to the result of the near-field measurement of the one-dimensional sound intensity probe. The proposed spectral separation method of the sound power of multiple sound sources provides a new method for accurate noise identification in industrial environments.

## 1. Introduction

The operation of mechanical equipment is often accompanied by strong noise, which contains a wealth of information about the status of equipment parts. If each sound source inside the machine can be separated, it is of great significance for monitoring the equipment state through noise [1]. In rotating machinery, the sound sources are mainly the rotating parts. Due to the transmission relationship, the noise signal usually contains same-frequency components. Therefore, the noise separation method must have the ability of accurate intensity separation for sounds of the same frequency.

In the field of signal processing, there are many ways to separate signals. The amplitude of each signal source obtained by blind signal separation is uncertain—that is, it is difficult to obtain the accurate strength of each source. The signal sources separated by this method need to be independent of each other, and sources with same-frequency components cannot be separated [2]. Empirical mode decomposition is based on the time-scale characteristics of the data [3], and methods such as wavelet decomposition are based on the frequency characteristics of the signal [4]. The components obtained by those methods are not directly related to the actual signal sources. Thus, these methods are difficult to apply to the separation of multiple sound sources in rotating machinery.

Among the sound source identification methods, near-field acoustic holography uses a microphone array to accurately reconstruct the sound pressure, particle velocity, and sound intensity of the three-dimensional sound field. It can realize the accurate calculation of the position and intensity of a sound source. However, the measurement array must be very close to the surface of the sound sources and cover the surface of the sound sources, which is very difficult for on-site measurement [5]. Acoustic beamforming is a signal processing technique based on far-field microphone array measurements. It collects the information of the sound field through the microphone array and then uses the beamforming algorithm to filter so that the sound source can be identified at a certain distance from the microphone array [6]. When the sound field environment is complex, the beamforming method only reflects the sound pressure contribution of the sound source in the microphone space plane and cannot calculate the accurate intensity of the sound source [7]. Sound source identification technology has a wide range of applications in life. In the field of classic industrial noise, Chu Z. et al. proposed a self-spectral mutually exclusive cross-spectral beamforming algorithm based on the spherical wave hypothesis. The improved algorithm was used to test the sound insulation performance of a car. The weak part of the acoustic seal was accurately identified [8]. In terms of vehicle noise source identification, in 2016, clustering inverse beamforming technology was used for the first time in vehicle sound source location. This method can improve the accuracy of sound source localization and recognition when the closed acoustic cavity is used as a carriage [9]. In the study of noise source identification in a port area, Fredianelli, L. et al. described the sound power level and 1/3 octave sound power spectrum of low-speed ships and identified five different types of noise emitted by ocean-going ships [10]. They also used a multiple regression analysis to analyze the impact of a ship’s minimum distance, speed, and draft on ship noise emissions [11].

Three-dimensional sound intensity technology is developed on the basis of one-dimensional sound intensity tests. A special three-dimensional sound intensity probe is used to obtain a spatial sound intensity vector and then analyze the characteristics of the sound source [12]. In 2009, Basten et al. used a single three-dimensional sound intensity probe to accurately locate two unrelated sound sources [13]. In 2009, Wind et al. used the same method to test the ability of two probes to locate one to five unrelated sound sources [14]. Jing et al. used beamforming algorithms to compare the localization capabilities of a single three-dimensional sound intensity probe and four common microphone arrays for a single sound source. The three-dimensional sound intensity probe can achieve a positioning accuracy similar to that of the microphone array, but the spatial resolution has nothing to do with the frequency [15]. In 2015, Kotus used a single probe to test single-frequency noise, pink noise, and actual impulse sound in an anechoic room. If the sound source spectrum in the analysis signal section does not overlap, high positioning accuracy can be achieved [16]. In 2020, Lu Yi et al. proposed a method of using multiple three-dimensional sound intensity probe arrays for sound source identification. The method can identify the position and power of multiple sound sources with same-frequency components in a three-dimensional space [17] and provides a new idea for the separation of mechanical noise sources.

Based on the method of using three-dimensional sound intensity probes to identify sound sources, this paper extends the method to the frequency spectrum separation of multiple sound sources.

## 2. Basic Principles of the Three-Dimensional Sound Intensity Measurement

**Figure 1:**
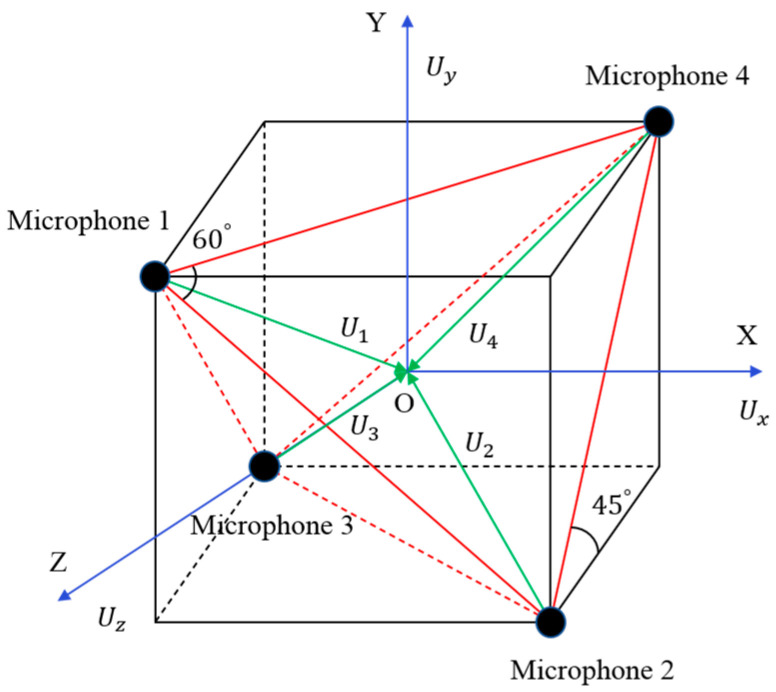
The schematic of the three-dimensional sound intensity probe.

The four microphones of the three-dimensional sound intensity probe were placed at the four vertices of a cube [18], as shown in Figure 1. First, we calculated the sound intensity components from the center of the cube to the vertices where the four microphones were located according to the dual-microphone cross-spectrum method. Then, we performed vector calculations according to the geometric characteristics of the cube and obtained the sound intensity components in the X, Y, and Z directions of the center point of the cube [19,20].

In Figure 1, the diagonal length of the cube surface is $d_{1}$, and the distance from each vertex to the center O is a, $a=(\sqrt{3}/2\sqrt{2})d_{1}$. Among the 4 microphones, the connection of any 3 microphones can form an equilateral triangle. $\vec{U_{x}(t)}$, $\vec{U_{y}(t)}$, and $\vec{U_{Z}\left(t\right)}$ are the particle velocities in the X, Y, and Z axes of the three-dimensional coordinate system, respectively. Supposing the sound pressure measured by the four microphones is $p_{1}(t)$, $p_{2}(t)$, $p_{3}(t)$, and $p_{4}(t)$ and their Fourier transform is $P_{1}(\omega )$, $P_{2}(\omega )$, $P_{3}(\omega )$, and $P_{4}(\omega )$, abbreviated as $P_{1}$, $P_{2}$, $P_{3}$, and $P_{4}$. Then, the sound pressure $P_{O}$ at the center point O is approximately equal to the average of the sound pressure measured by the four microphones, namely:

(1) $$P_{O}=\left(P_{1}+P_{2}+P_{3}+P_{4}\right)/4$$

According to the sound intensity measurement of the cross-power spectrum method, for a one-dimensional sound intensity probe composed of two microphones, the sound intensity at the center is:

(2) I(ω)→=12Re[P(ω)⋅U*(ω)→]=Im(G12)ρωΔr

In the formula, P(ω) and $\vec{U\left(\omega \right)}$ are the Fourier transform of the sound pressure P(t) and the particle velocity $\vec{U\left(t\right)}$ at the center point, respectively; Re is the real part of the complex number, and Im is the imaginary part. The superscript “*” indicates the conjugate of the complex number. $G_{12}$ is the cross-power spectrum on the sound pressure of the two microphones, and Δr is the distance between the microphones.

As shown in Figure 1, the vector synthesis of the four particle velocities from the four microphones to the center O corresponds to the total particle vibration velocity at the center O. By decomposing the particle vibration velocity into 3 coordinate axes, we can obtain:

(3) $$\vec{U_{x}}=\frac{\sqrt{3}}{4}(\vec{U_{1}}+\vec{U_{3}}-\vec{U_{2}}-\vec{U_{4}})$$

(4) $$\vec{U_{y}}=\frac{\sqrt{3}}{4}(\vec{U_{2}}+\vec{U_{3}}-\vec{U_{1}}-\vec{U_{4}})$$

(5) $$\vec{U_{z}}=\frac{\sqrt{3}}{4}(\vec{U_{4}}+\vec{U_{3}}-\vec{U_{2}}-\vec{U_{1}})$$

Taking Equation (2) as a reference, we can obtain the sound intensity vector in the X direction at the center point O as:

(6) Ix(ω)→=12Re[P(ω)⋅Ux*(ω)→]=(2/4ωρd1)⋅Im[G12+G32+G14+G34]

In Equation (6), $\vec{U_{x}\left(\omega \right)}$ is the particle velocity in the X direction and $G_{ij}$ is the cross-power spectrum of the i-th microphone and the j-th microphone signals.

Similarly, the sound intensity vector in the Y direction at the center point O is:

(7) Iy(ω)→=12Re[P(ω)⋅Uy*(ω)→]=(2/4ωρd1)⋅Im[G21+G31+G24+G34]

The sound intensity vector in the Z direction at the center point O is:

(8) Iz(ω)→=12Re[P(ω)⋅Uz*(ω)→]=(2/4ωρd1)⋅Im[G31+G41+G42+G32]

According to Equations (6)–(8), the sound intensity component at the center point O can be calculated from the sound pressure on the 4 microphones. The total sound intensity vector is composed of components in the X, Y, and Z directions. If there are multiple sound sources, the measured sound intensity vectors are the synthesis of multiple sound sources, and the intensity of each sound source cannot be obtained. However, if the number of three-dimensional sound intensity probes is increased and the sound field is sampled at multiple points, it is possible to completely determine the sound power spectrum of the sound source. Lu Yi used this method to identify the position and intensity of two sound sources by three three-dimensional sound intensity probes and showed that this method has high accuracy.

## 3. The Separation of the Sound Power Spectrum of Multiple Sound Sources

For N point sources, the coordinates in the spherical coordinate system are $\vec{P_{Sn}}=(\rho_{Sn},\;\alpha_{Sn},\;\theta_{Sn})$, n=1, 2, …, N, and the corresponding sound power is $W_{n}$, n=1, 2, …, N. *M* three-dimensional sound intensity probes are used for the measurement, and the probe coordinates are $\vec{P_{Tm}}=(\rho_{Tm},\;\alpha_{Tm},\;\theta_{Tm})$, m=1, 2, …, M, respectively. Then, the path vector from the sound source n to the probe m is $\vec{P_{Tm}}-\vec{P_{Sn}}$.

According to the propagation law of the monopole sound source in the free field, without considering the actual environmental factors such as scattering, the sound intensity generated by the sound source n on the probe m is:

(9) $$\vec{I_{SnTm}\left(\omega \right)}=\frac{W_{n}\left(\omega \right)}{4\pi {\left(|\vec{P_{Tm}}-\vec{P_{Sn}}|\right)}^{2}}∠(\vec{P_{Tm}}-\vec{P_{Sn}})$$

The total sound intensity measured on each probe is the superposition of the sound intensities produced by each of the N sound sources, which is:

(10) $$\vec{I_{Tm}\left(\omega \right)}=\sum_{n=1}^{n=N}\vec{I_{SnTm}\left(\omega \right)}=\sum_{n=1}^{n=N}\frac{W_{n}\left(\omega \right)}{4\pi {\left(|\vec{P_{Tm}}-\vec{P_{Sn}}|\right)}^{2}}∠(\vec{P_{Tm}}-\vec{P_{Sn}})$$

When the positions of sound sources and probes are known, Equation (10) constitutes a linear equation system for measuring sound intensity and sound power, since the sound intensity is vectoral and the m vectors can be decomposed into a spatial rectangular coordinate system to obtain 3M equations. In theory, as long as the number of probes is no less than 1/3 of the number of sound sources, the equation set can be solved directly to obtain the sound power of each sound source on each frequency (i.e., $W_{n}\left(\omega \right)$). However, in practice, due to the fact that the sound sources are not ideal monopole sources as well as directionality of intensity radiation, sound field environment, and noise interference, it is more feasible to solve the optimal solution of the equations by numerical methods.

Particle swarm optimization (PSO) is used to solve the 3M equations decomposed from Equation (10). The authors of the particle swarm optimization algorithm are Kennedy and Eberhart [21]. The characteristics of this algorithm are random search and parallel optimization. The algorithm can be well adapted to engineering applications [22]. The basic concept of PSO comes from the study of the foraging behavior of bird flocks. It uses particles to imitate the social behavior of individual birds when searching for food and searches for the global optimum in parallel in a multi-dimensional space [23]. In multi-dimensional space, each particle can be regarded as a search individual, the current position of the particle is the candidate solution of the corresponding optimization problem, and the flight process of the particle is the search process of the search individual. The optimal individual extreme value in the particle swarm is taken as the current global optimal value. In the iterative process, the particles update their velocity and position through individual optimal values and global optimal values.

The iterative formula of particle velocity and position in particle swarm algorithm is:

(11) $$V_{i}^{d}=\omega \times v_{i}^{d}+c_{1}\times r_{1}\times (p_{i}^{d}-x_{i}^{d})+c_{2}\times r_{2}\times (p_{g}^{d}-x_{i}^{d})$$

(12) $$X_{i}^{d}=x_{i}^{d}+\alpha \times v_{i}^{d}$$

where i(i=1, 2, 3, …, m) is the particle number; d(d=1, 2, 3, …, D) is the dimension number; $X_{i}=(x_{i}^{1},\;x_{i}^{2},\;\ldots ,\;x_{i}^{D})$ is a solution of the optimization problem corresponding to the position of the i-th particle in the particle swarm; $P_{i}=(p_{i}^{1},\;p_{i}^{2},\;\ldots ,\;p_{i}^{D})$ is the optimal position at all distances experienced by the i-th particle; $P_{g}=(p_{g}^{1},\;p_{g}^{2},\;\ldots ,\;p_{g}^{D})$ is the optimal position at all distances experienced by all particles; $V_{i}=(v_{i}^{1},\;v_{i}^{2},\;\ldots ,\;v_{i}^{D})$ is the search speed of particle i; ω is the non-negative inertia factor, $c_{1}$ and $c_{2}$ are non-negative acceleration constants; $r_{1}$ and $r_{2}$ are random numbers in the range of 0 to 1; α is the constraint factor to control the weight of speed.

The process flow of the PSO algorithm is as follows:

(1) Initialize the particle swarm, including the population size (set to 100), inertia weight ω (set to 0.1), acceleration constant $c_{1}$ (set to 2.2), $c_{2}$ (set to 2.1), and the random position and the search speed of each particle (set the maximum speed to 3 m/s).
(2) Calculate the objective function that is the fitness function.
(3) Update the individual optimal value of the particle and the global optimal value of the group according to the fitness function value and update the optimal position and optimal speed of the particle according to Equations (11) and (12).
(4) Determine whether the maximum number of iterations (set to 1000) or the global optimal position is reached and whether the minimum limit is satisfied. If satisfied, end the iteration; otherwise, repeat steps 2–4.

The same parameters were used both in the experiment and in the simulation. Since both the sound intensity $I_{Tm}$ and the sound power $W_{n}$ are the function of the frequency, the particle swarm algorithm can be used to solve Equation (10) at each frequency point of the spectrum to obtain the sound power at each frequency point. It is equivalent to the power spectral density of sound power, and the frequency spectrum separation of each sound source can be realized.

## 4. Simulation Analysis

A simulation experiment of two sound sources measured with one three-dimensional sound intensity probe was established. The spherical coordinates of the three-dimensional intensity probe were $\vec{P_{T}}=(0,\;0{}^{\circ},\;90{}^{\circ})$. The coordinates of sound source 1 were $\vec{P_{S1}}=(1.73,\;45{}^{\circ},\;54.7{}^{\circ})$, and for sound source 2 were $\vec{P_{S2}}=(1.12,\;0{}^{\circ},\;63.5{}^{\circ})$. Both sound sources contained three frequencies of 500, 1000, and 2000 Hz. In sound source 1, the sound power at 500 Hz was 0.00605 W, the sound power at 1000 Hz was 0.02 W, and the sound power at 2000 Hz was 0.0128 W. The sound power of the three frequencies in the sound source 2 was 0.08, 0.0072, and 0.03125 W, respectively.

We substituted the signals on the four microphones into Equations (6)–(8) to calculate the sound intensity values in the X, Y, and Z directions of the probe, as shown in Table 1.

**Figure 2:**
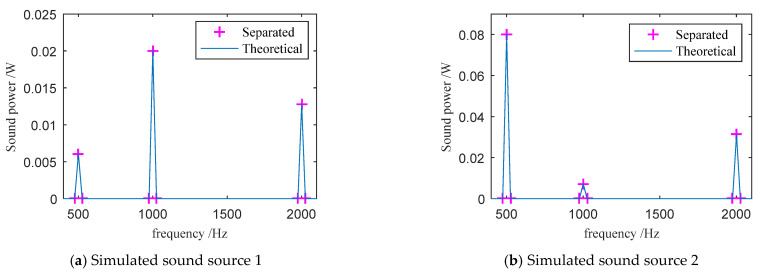
Comparison of the separated sound power spectra and the theoretical values.

We then substituted the above position and sound intensity into Equation (10) to solve the sound power of the sound source. According to the calculation results, the sound power spectra of the two sound sources were separated, as shown in Figure 2. It can be seen that the separated sound power spectrum is consistent with the theoretical sound power spectrum of the simulated sound source, and there is no obvious error.

## 5. Experimental Verification

In order to verify the feasibility of the three-dimensional sound intensity vector decomposition method for spectrum separation in the actual environment, experimental research was carried out on the QPZZ-II fault simulation test bench in an ordinary laboratory environment. The experimental bench was located at the corner of the laboratory, and the distances to the two walls were 940 and 610 mm. The nearby walls were treated with simple sound absorption. The speed of the motor was 888.75 r/min and the shaft frequency was 888.75/60 = 14.8125 Hz.

**Figure 3:**
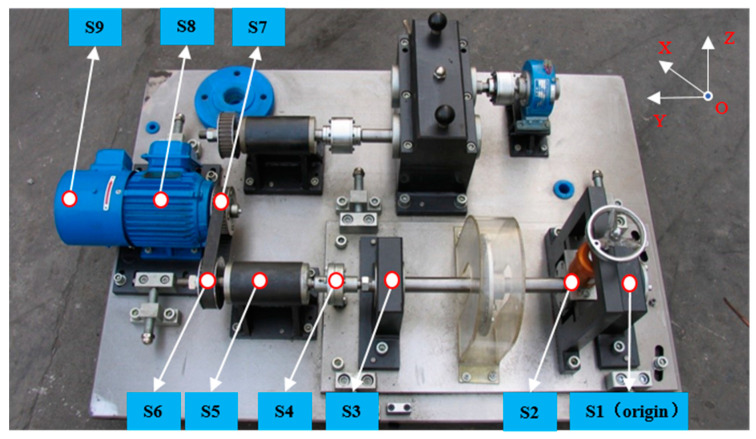
The nine sound sources on the test bench.

As shown in Figure 3, the sound sources on the test bench included motors, pulleys, couplings, and various support bearings. The vertical distance between the probe and the transmission shaft plane was 500 mm. Since the distance between the center of the nine sound sources and the test point was more than two times the maximum geometric size of the sound sources, the nine sound sources were all regarded as point sound sources. The spherical coordinate system was set up with the axial position of bearing seat I (S1) as the origin. The specific information of the sound sources is shown in Table 2.

In order to obtain the true strength of each sound source, the one-dimensional sound intensity probe was used to collect the intensity of each source at a distance of 30 mm from the centers of each sound source.

Since the one-dimensional sound intensity probe was close to the sound source and pointed to the sound source, the influence of other sound sources can be ignored according to the characteristics of sound intensity. Based on the point sound source hypothesis, the sound power spectrum of each sound source can be obtained referring to Equation (9). This sound power spectrum is taken as the ground truth to verify the proposed method. 

Since there were nine sound sources, three three-dimensional sound intensity probes were used for testing. Four MPA416 array microphones were installed on each probe. A dynamic signal instrument was used to collect the microphone signals, and the sampling frequency was 25.6 kHz. The vertical distance between the probe and the transmission shaft plane was 500 mm. Probe 1 was located above the loading device (sound source S6), and the spherical coordinates were $\vec{P_{T1}}=(93,\;90{}^{\circ},\;57{}^{\circ})$. Probe 2 was located above bearing seat II (sound source S3), and the coordinates were $\vec{P_{T2}}=(67,\;90{}^{\circ},\;41{}^{\circ})$. Probe 3 was located above pulley I (sound source S6), with the coordinates $\vec{P_{T3}}=\left(51,\;90{}^{\circ},\;9.0{}^{\circ}\right)$.

**Figure 4:**
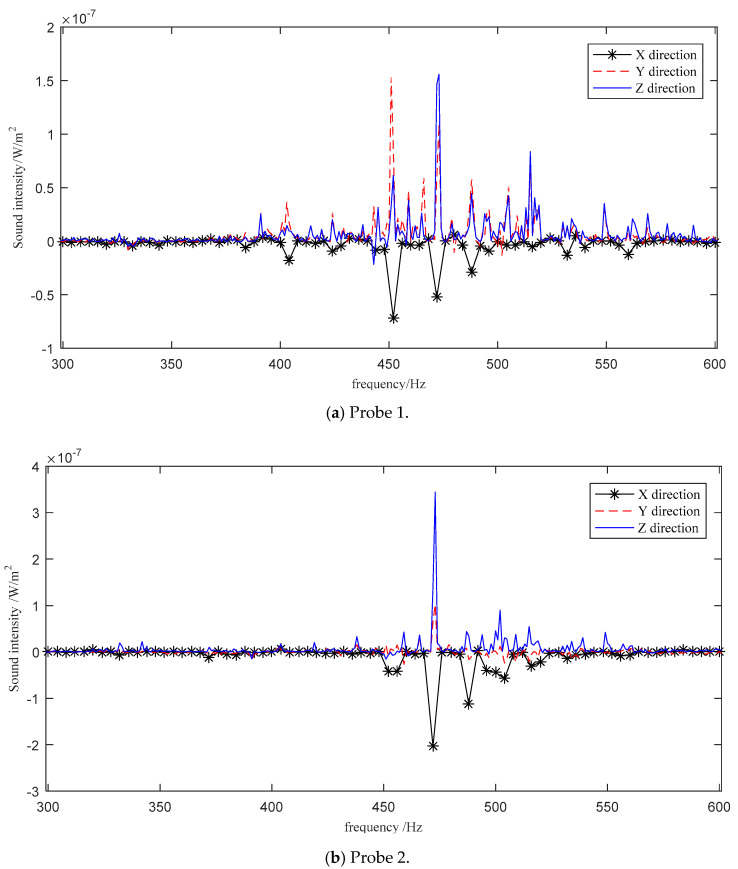

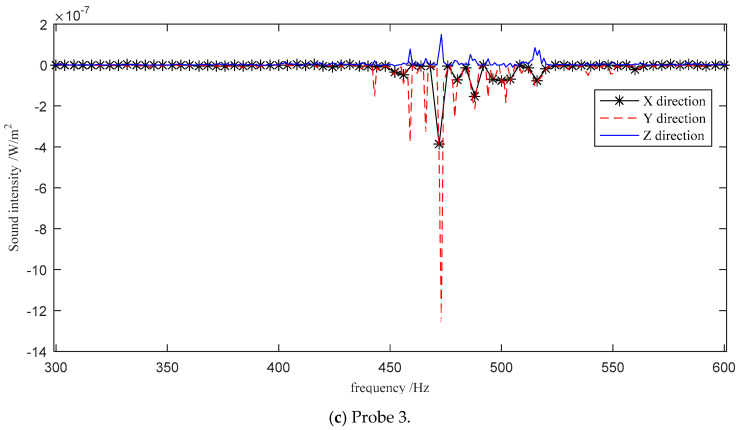
Sound intensity measured by the three-dimensional probe.

The sound intensity spectra of the main frequency bands measured by three three-dimensional sound intensity probes are shown in Figure 4. We selected the frequency point with a sound power value greater than 1 × 10^−13^ W for sound power spectrum separation. The reasons are that firstly, the sound power value at other frequency points is too small, and the contribution value to the sound power spectrum is not large; secondly, the amount of calculations can be reduced.

**Figure 5:**
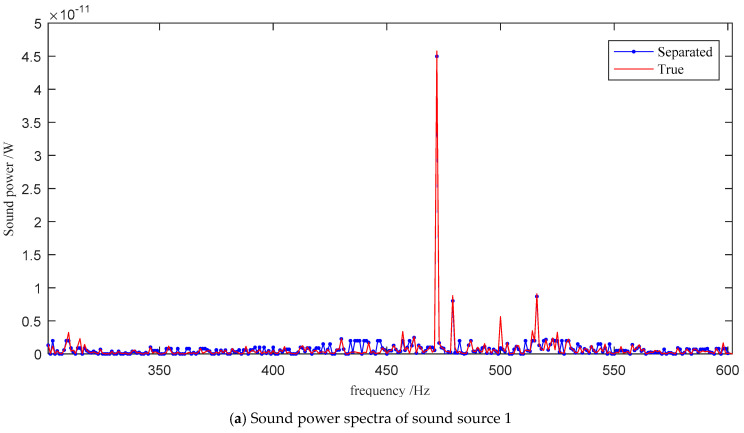

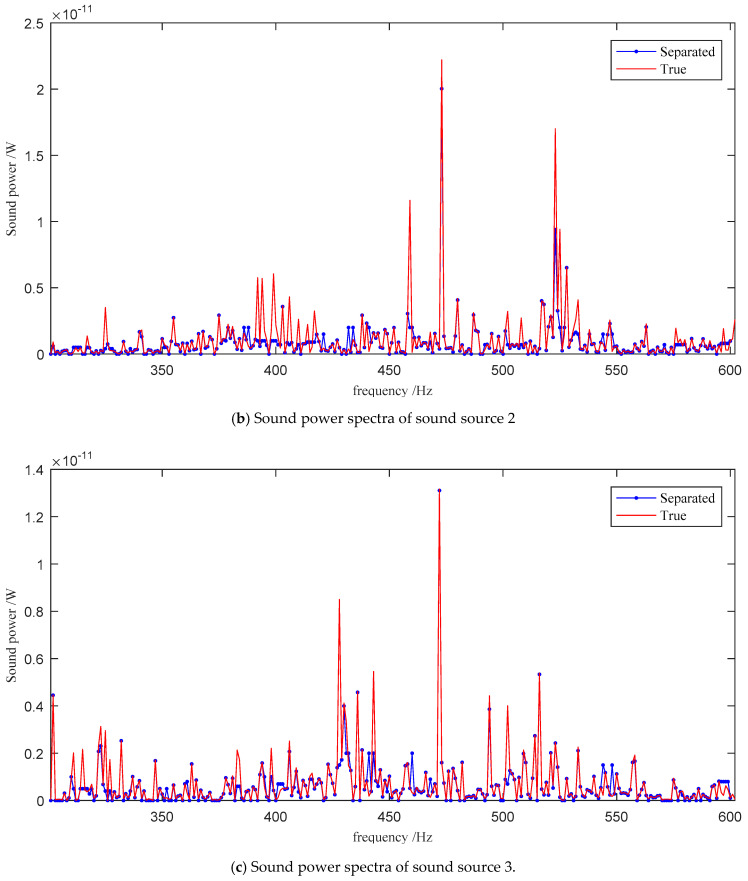

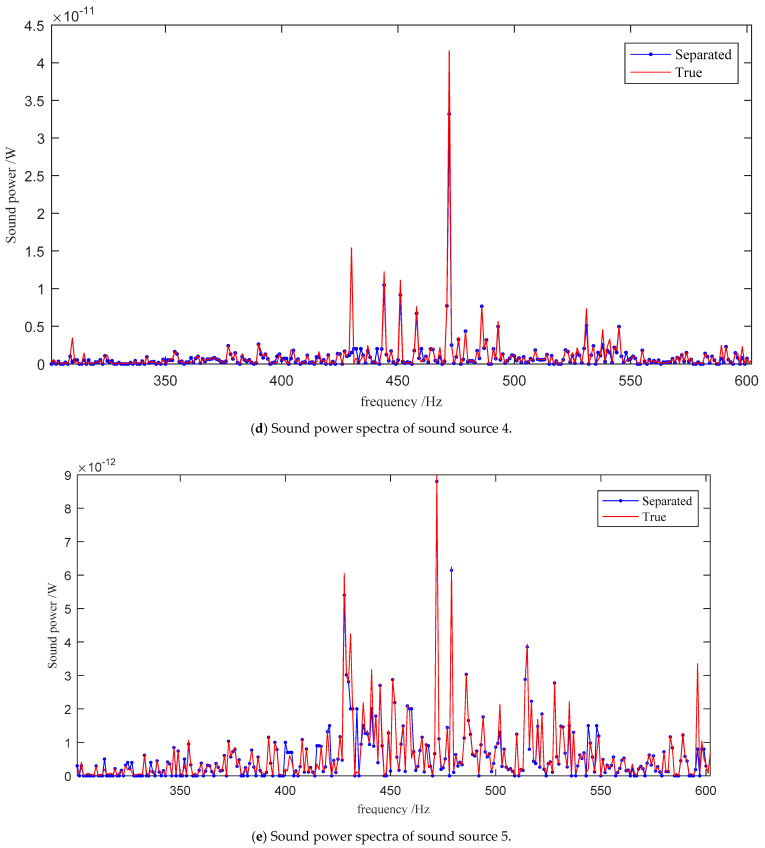

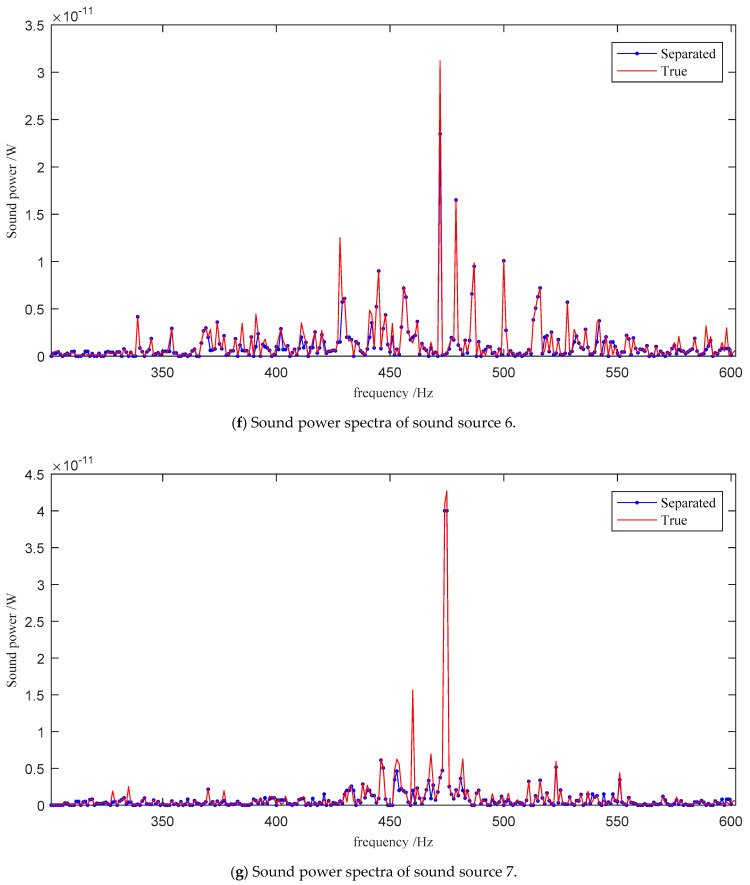

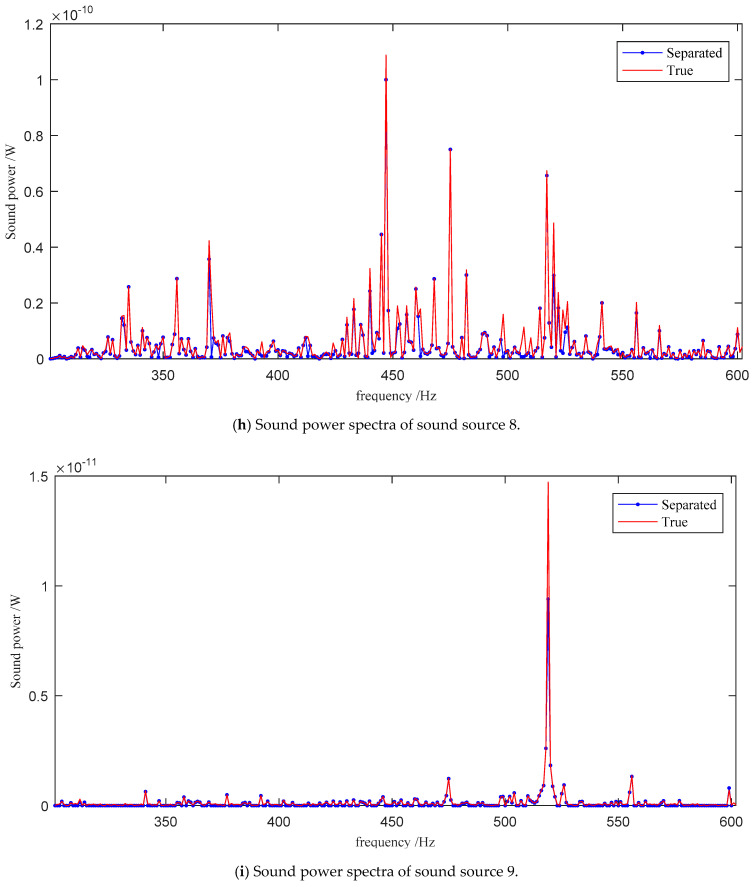
The comparison of the separated and the true sound power spectra of the nine sound sources.

The positions of the sound sources, the positions of the probes, and the sound intensity were substituted into Equation (10) as known quantities, and the PSO was used to solve the sound power of the nine sound sources at each frequency point. The separated spectra were compared with the true power spectra measured by the one-dimensional sound intensity probe, as shown in Figure 5.

The sound power spectra of the nine sound sources calculated by the sound intensity vector decomposition method are very close to the true values, and the errors are relatively small at the frequencies with higher intensity.

The dominating frequency in the sound sources S1–S6 is 472 Hz, and the recognition errors are all small. The specifications of the two pulleys are the same, and the number of teeth is 32. The meshing frequency of the toothed belt is equal to the product of the shaft frequency and the number of teeth, and the meshing frequency of the ruler belt is 474 Hz. Here, 472 Hz is close to the meshing frequency of the toothed belt. The six sound sources are distributed on two rigidly connected slender shafts, thus sharing the same peak frequency. The power on S1, S4, and S6 is greater than on the other three sources. S1 is the test bearing of the test bench, which is often disassembled and assembled, and the bearing clearance is large. S4 is the coupling position, and there may be a matching problem. S6 is pulley I, which is directly excited by the meshing of the belt and the gear teeth.

For the sound source S7, the dominating frequency is 475 Hz. S7 is the pulley on the driving side. Compared with the pulley S6 on the load side, the peak frequency shifted slightly.

The dominating frequency of S8 is 447 Hz. S8 is at the center of the motor, and the sound intensity is the highest among all sources. The frequency spectrum structure is more complicated. The sound source S9 is a motor fan with five blades and its peak frequency is 519 Hz, which is seven times the blade-passing frequency of 74 Hz.

It can be seen that the three-dimensional sound intensity vector decomposition method can recover the intensity of the main frequency range of each sound source accurately, and the result is equivalent to that of one-dimensional sound intensity measured at close distance. The recovery ratio *Rr* is used to quantify the accuracy. *Rr* is the proportion of the separated sound power $W_{s}^{\prime }\left(\omega \right)$ and the true sound power $W_{t}^{\prime }\left(\omega \right)$, as:

(13) $$R_{r}=\frac{\sum \omega W_{s}^{\prime }\left(\omega \right)}{\sum \omega W_{t}^{\prime }\left(\omega \right)}$$

To omit the background noise, $W_{s}^{\prime }\left(\omega \right)$ and $W_{t}^{\prime }\left(\omega \right)$ were selected from the sound power spectra with values greater than 1 × 10^−12^ W.

(14) $$W_{s}^{\prime }\left(\omega \right)=\left\{ \begin{array}{l} W_{s}\left(\omega \right),W_{s}\left(\omega \right)>10^{-12}\\ 0,W_{s}\left(\omega \right)\leq 10^{-12} \end{array}\right.$$

(15) $$W_{t}^{\prime }\left(\omega \right)=\left\{ \begin{array}{l} W_{t}\left(\omega \right),W_{t}\left(\omega \right)>10^{-12}\\ 0,W_{t}\left(\omega \right)\leq 10^{-12} \end{array}\right.$$

where $W_{s}\left(\omega \right)$ is the sound power spectrum separated by the proposed method, and $W_{t}\left(\omega \right)$ is the true sound power spectrum calculated from the near-field sound intensity measurement.

**Figure 6:**
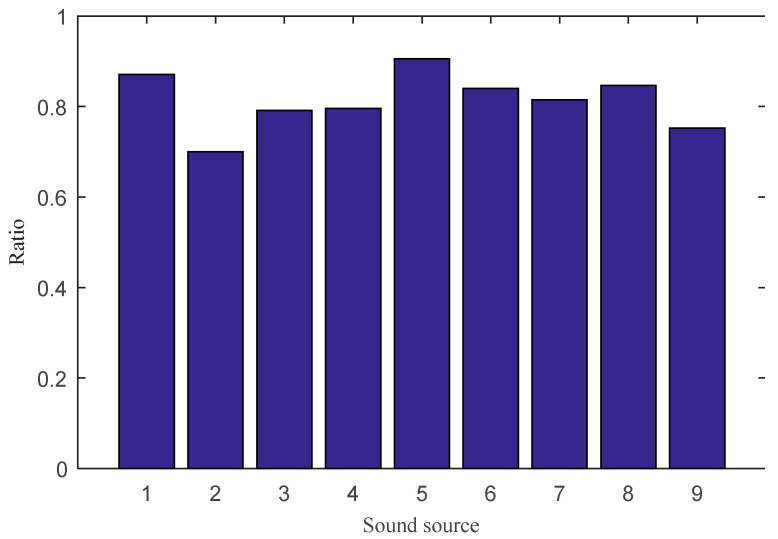
The recovery ratios of the nine sound sources in the experiment.

The recovery ratio Rr values of the nine sources are shown in Figure 6. The closer this ratio is to 1, the closer the separation sound power value is to the true value and the smaller the separation error. The highest value is 0.9 for source 5, while the lowest is 0.7 for source 2.

## 6. Conclusions

Based on the three-dimensional sound intensity measurement method, this paper proposes the sound power spectrum separation method for multiple sound sources. Given the positions of the sound sources, by solving the equations of the three-dimensional sound intensity on the sound power of the sources, the sound power of each sound source at each frequency is calculated. Thus, the sound power spectrum of each sound source is obtained. Taking the traditional near-field one-dimensional sound intensity measurement as the reference, the main frequency bands of multiple sound sources on the test bench were separated in an ordinary laboratory, and the accuracy of the three-dimensional sound intensity vector decomposition spectrum separation method was verified.

Compared with other sound source identification methods, this method can separate the sound power spectra of multiple noise sources over a longer distance, providing a new method for noise monitoring and diagnosis of rotating machinery. However, there are some shortcomings. First, it is assumed that the sound sources are ideal point sound sources, but in fact, the sound source may have a certain volume or have multiple types, which will also affect the coordinates of the sound sources to a certain extent. Furthermore, there are interfering sound sources in an ordinary laboratory, but we ignored the noise interference from the external environment. In addition, when there are too many sound sources or too many frequency components, too much calculation may reduce the calculation accuracy. In the future, further research will be conducted from the above-mentioned aspects.

## Figures and Tables

**Table 1:** The simulated sound intensity in three directions.

Frequency	$I_{Tx}\left(W/m^{2}\right)$	$I_{Ty}\left(W/m^{2}\right)$	$I_{Tz}\left(W/m^{2}\right)$
500 Hz	−0.0082	0.0889	−6.5578 × 10^−5^
1000 Hz	−0.0272	0.0404	−2.1679 × 10^−4^
2000 Hz	−0.0174	0.0521	−1.3874 × 10^−4^

**Table 2:** The locations of the nine sound sources.

Number	Sound Source	Coordinates (mm,°,°)
S1	The axis of bearing seat I	$\vec{P_{S1}}=(0,\;0{}^{\circ},\;0{}^{\circ})$
S2	The axis of the loading device	$\vec{P_{S2}}=(8,\;90{}^{\circ},\;90{}^{\circ})$
S3	The axis of bearing seat II	$\vec{P_{S3}}=(44,90{}^{\circ},90{}^{\circ})$
S4	The axis of the rigid coupling	$\vec{P_{S4}}=(55,\;90{}^{\circ},\;90{}^{\circ})$
S5	The axis of the midpoint of the bearing support	$\vec{P_{S5}}=(66.5,\;90{}^{\circ},\;90{}^{\circ})$
S6	The axis of the pulley I	$\vec{P_{S6}}=(78,\;90{}^{\circ},\;90{}^{\circ})$
S7	The axis of the pulleyII	$\vec{P_{S7}}=(80.1,\;77{}^{\circ},\;88.2{}^{\circ})$
S8	The rotor in the middle of the motor	$\vec{P_{S8}}=(96,\;79{}^{\circ},\;91{}^{\circ})$
S9	The fan at the rear of the motor	$\vec{P_{S9}}=(111,\;81{}^{\circ},\;91{}^{\circ})$

## Data Availability

The data presented in this study are available on request from the corresponding author.
